# Emergence of an Extensively Drug-Resistant *Salmonella enterica* Serovar Typhi Clone Harboring a Promiscuous Plasmid Encoding Resistance to Fluoroquinolones and Third-Generation Cephalosporins

**DOI:** 10.1128/mBio.00105-18

**Published:** 2018-02-20

**Authors:** Elizabeth J. Klemm, Sadia Shakoor, Andrew J. Page, Farah Naz Qamar, Kim Judge, Dania K. Saeed, Vanessa K. Wong, Timothy J. Dallman, Satheesh Nair, Stephen Baker, Ghazala Shaheen, Shahida Qureshi, Mohammad Tahir Yousafzai, Muhammad Khalid Saleem, Zahra Hasan, Gordon Dougan, Rumina Hasan

**Affiliations:** a Wellcome Trust Sanger Institute, Hinxton, United Kingdom; b The Aga Khan University, Karachi, Pakistan; c University of Cambridge Department of Medicine, Cambridge, United Kingdom; d Gastrointestinal Bacteria Reference Unit, National Infection Service, Public Health England, London, United Kingdom; e The Hospital for Tropical Diseases, Wellcome Trust Major Overseas Programme, Oxford University Clinical Research Unit-Vietnam, Ho Chi Minh City, Vietnam; f Centre for Tropical Medicine and Global Health, Oxford University, Oxford, United Kingdom; g Faculty of Infectious and Tropical Diseases, London School of Hygiene and Tropical Medicine, London, United Kingdom; Emory University

**Keywords:** antibiotic resistance, *Salmonella*, Typhi, typhoid

## Abstract

Antibiotic resistance is a major problem in *Salmonella enterica* serovar Typhi, the causative agent of typhoid. Multidrug-resistant (MDR) isolates are prevalent in parts of Asia and Africa and are often associated with the dominant H58 haplotype. Reduced susceptibility to fluoroquinolones is also widespread, and sporadic cases of resistance to third-generation cephalosporins or azithromycin have also been reported. Here, we report the first large-scale emergence and spread of a novel *S*. Typhi clone harboring resistance to three first-line drugs (chloramphenicol, ampicillin, and trimethoprim-sulfamethoxazole) as well as fluoroquinolones and third-generation cephalosporins in Sindh, Pakistan, which we classify as extensively drug resistant (XDR). Over 300 XDR typhoid cases have emerged in Sindh, Pakistan, since November 2016. Additionally, a single case of travel-associated XDR typhoid has recently been identified in the United Kingdom. Whole-genome sequencing of over 80 of the XDR isolates revealed remarkable genetic clonality and sequence conservation, identified a large number of resistance determinants, and showed that these isolates were of haplotype H58. The XDR *S*. Typhi clone encodes a chromosomally located resistance region and harbors a plasmid encoding additional resistance elements, including the *bla*_CTX-M-15_ extended-spectrum β-lactamase, and carrying the *qnrS* fluoroquinolone resistance gene. This antibiotic resistance-associated IncY plasmid exhibited high sequence identity to plasmids found in other enteric bacteria isolated from widely distributed geographic locations. This study highlights three concerning problems: the receding antibiotic arsenal for typhoid treatment, the ability of *S*. Typhi to transform from MDR to XDR in a single step by acquisition of a plasmid, and the ability of XDR clones to spread globally.

## IMPORTANCE

Typhoid fever is a severe disease caused by the Gram-negative bacterium *Salmonella enterica* serovar Typhi. Antibiotic-resistant *S*. Typhi strains have become increasingly common. Here, we report the first large-scale emergence and spread of a novel extensively drug-resistant (XDR) *S*. Typhi clone in Sindh, Pakistan. The XDR *S*. Typhi is resistant to the majority of drugs available for the treatment of typhoid fever. This study highlights the evolving threat of antibiotic resistance in *S*. Typhi and the value of antibiotic susceptibility testing and whole-genome sequencing in understanding emerging infectious diseases. We genetically characterized the XDR *S*. Typhi to investigate the phylogenetic relationship between these isolates and a global collection of *S*. Typhi isolates and to identify multiple genes linked to antibiotic resistance. This *S*. Typhi clone harbored a promiscuous antibiotic resistance plasmid previously identified in other enteric bacteria. The increasing antibiotic resistance in *S*. Typhi observed here adds urgency to the need for typhoid prevention measures.

## INTRODUCTION

Typhoid fever remains a significant public health threat in low- and middle-income countries, with an estimated ~200,000 typhoid-associated deaths each year (1). Typhoid fever is caused by the bacterial pathogen *Salmonella enterica* subsp. *enterica* serovar Typhi (*S*. Typhi), a human-restricted monophyletic serovar of *S. enterica*. *S*. Typhi is transmitted from human to human by the fecal-oral route, often via contaminated water. Vaccination, access to clean water, and improved sanitation are effective means to prevent typhoid. Antibiotics are also vital to the treatment of typhoid, but antibiotic-resistant *S*. Typhi strains have become increasingly prevalent.

Historically, the first-line treatments for typhoid have been ampicillin, trimethoprim-sulfamethoxazole, and chloramphenicol (2). *S*. Typhi strains with resistance to these three antibiotics are considered multidrug resistant (MDR), and such isolates were first observed in the late 1970s to early 1980s. Resistance to the second-line antibiotics the fluoroquinolones has also been frequently reported since these became the preferred treatment in regions with MDR infections. Ceftriaxone, a third-generation cephalosporin, and azithromycin, a macrolide, are now also used to treat typhoid fever when other options cannot be used (2). However, sporadic cases of ceftriaxone- or azithromycin-resistant *S*. Typhi have recently been reported.

Over the past two decades, a dominant, commonly MDR, haplotype of *S*. Typhi called H58 has been spreading globally (3). It is prevalent across South and Southeast Asia and parts of Africa and Oceania. Multiple local outbreaks of typhoid have been linked to various sublineages of H58 (4–7).

The transfer of antimicrobial resistance (AMR) genes between bacteria is commonly facilitated by plasmid or transposon exchange. In H58, as with other *S*. Typhi clades, the AMR genes are generally associated with an IncHI1 plasmid. Such plasmids harbor a composite transposon that can carry multiple resistance genes, including *bla*_TEM-1_ (ampicillin resistance), *dfrA7*, *sul1*, *sul2* (trimethoprim-sulfamethoxazole resistance), *catA1* (chloramphenicol resistance), and *strAB* (streptomycin resistance) genes. This composite transposon has also been found integrated into the chromosome in some H58 *S*. Typhi lineages (3, 5). Ceftriaxone resistance, although previously uncommon in *S*. Typhi, is associated with the acquisition of an extended-spectrum β-lactamase (ESBL) gene.

Reduced susceptibility to fluoroquinolones is associated with chromosomal mutations and acquisition of AMR genes. In *S*. Typhi H58 lineages, mutations in the quinolone resistance-determining region (QRDR), comprised of the DNA gyrase (*gyrA* and *gyrB*) and topoisomerase IV (*parC* and *parE*) genes, are becoming common. The acquisition of plasmid-mediated quinolone resistance (PMQR) genes, such as *qnr*, *oqxAB*, or *aac(6′)Ib-cr*, can also contribute to fluoroquinolone resistance. Multiple QRDR single nucleotide polymorphisms (SNPs) or a combination of QRDR SNPs and PMQR genes results in fluoroquinolone resistance. Recently, fluoroquinolone treatment failure in typhoid patients was associated with three QRDR SNPs in Nepal (7).

In Pakistan, MDR and quinolone-resistant *S*. Typhi strains have been a major public health concern (8). While principal control efforts are directed to water, sanitation, and hygiene (WASH) measures, diagnosis and effective treatment of typhoid fever may contribute to control by potentially eliminating fecal carriers and shedders from the population. Since the emergence and spread of fluoroquinolone-nonsusceptible *S*. Typhi in Pakistan, the empirical treatment of choice for typhoid fever has been a third-generation cephalosporin such as ceftriaxone/cefotaxime (parenteral) or cefixime (oral). Laboratory surveillance data from Pakistan from 2009 to 2011 demonstrated the rise of MDR *S*. Typhi and a very small proportion of sporadic ceftriaxone resistance (0.08%, in 2 children from Karachi) (8).

Since November 2016, a large proportion of ceftriaxone-resistant cases have been identified in the province of Sindh, Pakistan, primarily from the cities of Hyderabad and Karachi. A similar case was also identified in the United Kingdom from a traveler returning from Pakistan. These *S*. Typhi strains were resistant to chloramphenicol, ampicillin, trimethoprim-sulfamethoxazole, fluoroquinolones, and third-generation cephalosporins, leaving limited treatment options. Here, we report the emergence and spread of these extensively drug-resistant (XDR) isolates as observed through positive blood cultures obtained from febrile patients. We used whole-genome sequencing (WGS) of over 80 isolates to comprehensively characterize the genetic basis of antibiotic resistance in this clonal population of H58 *S*. Typhi. We identified a plasmid potentially acquired from *Escherichia coli* that carries both an extended-spectrum β-lactamase (ESBL) gene and a *qnr* fluoroquinolone resistance gene. The emergence of this XDR *S*. Typhi clone highlights the need for urgent action before such lineages become the norm and it becomes more difficult to treat typhoid with existing drugs.

## RESULTS

Ceftriaxone-resistant typhoid fever cases were initially detected in November 2016 in Hyderabad, Pakistan. Following disc diffusion assays of the blood-isolated samples, the isolates were found to be resistant to ceftriaxone, ciprofloxacin, ampicillin, and trimethoprim-sulfamethoxazole and susceptible to imipenem, meropenem, and azithromycin. Three hundred thirty-nine isolates with the same XDR pattern were isolated from the Sindh region of Pakistan between November 2016 and September 2017 (see Fig. S1 in the supplemental material). The majority of the cases were located in Hyderabad and Karachi. Whole-genome sequencing was carried out on 87 of the XDR *S*. Typhi strains isolated in Sindh, Pakistan, over a 6-month period between November 2016 and March 2017. Twelve ceftriaxone-susceptible isolates collected from the same locations over an analogous time period were also sequenced for context (listed in Table S1). A complete reconstruction of the entire genome of a representative XDR isolate (22420_1_10_Pak60006_2016) was created using a combination of Nanopore and PacBio long-read sequencing methods. The final assembled chromosome was 4,733,003 bp in length along with a plasmid of 84,492 bp. This finished genome sequence was used as a reference for subsequent analyses.

10.1128/mBio.00105-18.1 FIG S1  Epidemiological data for XDR isolates in Sindh, Pakistan. Three hundred thirty-nine culture-confirmed ceftriaxone-resistant *S*. Typhi strains were isolated from the southern province of Sindh, Pakistan, between November 2016 and August 2017. (A) Geographic distribution of ceftriaxone-resistant cases. Map of Sindh province in Pakistan (inset). The largest numbers of cases were from the cities of Hyderabad (273) and Karachi (53). (B) Number of ceftriaxone-resistant isolates received by month. (C) Number of patients with ceftriaxone-resistant typhoid by age. Download FIG S1, PDF file, 0.9 MB. Copyright © 2018 Klemm et al. 2018 Klemm et al. This content is distributed under the terms of the Creative Commons Attribution 4.0 International license.

10.1128/mBio.00105-18.4 TABLE S1  Antibiogram data and accession numbers for samples sequenced in this study. Accession numbers, demographics in terms of city, results of identification, and antimicrobial susceptibilities of the isolates from this study. AMP, ampicillin; C, chloramphenicol; CRO, ceftriaxone; CIP, ciprofloxacin; CFM, cefuroxime; SXT, sulfamethoxazole; AZM, azithromycin; IPM, imipenem; FOT, fosfomycin; MEM, meropenem; ERT, ertapenem; AZM, azithromycin; KHI, Karachi; HYD, Hyderabad; NT, not tested. MIC breakpoints (micrograms per milliliter) according to CLSI guidelines given in publication M100:S27 (2017) were as follows: ciprofloxacin, susceptible, <0.06; intermediate, 0.12 to 0.5; resistant, >1; meropenem, susceptible, ≤1; intermediate, 2; resistant, ≥4; ertapenem, susceptible, ≤0.5; intermediate, 1; resistant, ≥2; azithromycin, susceptible, ≤16; resistant, ≥32. Download TABLE S1, XLSX file, 0.02 MB. Copyright © 2018 Klemm et al. 2018 Klemm et al. This content is distributed under the terms of the Creative Commons Attribution 4.0 International license.

**FIG 1:**
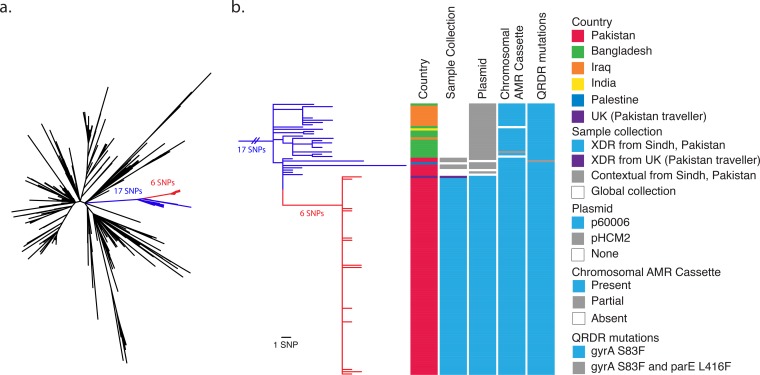
XDR isolates from Sindh, Pakistan, form a distinct cluster within the H58 phylogeny. (a) An unrooted maximum-likelihood phylogenetic tree of 98 Sindh, Pakistan, H58 isolates; 1 United Kingdom traveler isolate; and 853 global H58 isolates inferred from 1,920 SNPs using 22420_1_10_Pak60006_2016 as a reference. The XDR samples (red branches) are separated by 6 SNPs from the rest of the branch (blue), which is separated by 17 SNPs from the other H58 isolates. (b) A higher-resolution diagram of the branch from panel a rooted on nearest neighbor 10060_5_62_Fij107364_2012 displaying data for each isolate: country, sample collection (XDR or contextual organisms collected in Sindh, Pakistan, from 2016 to 2017; XDR organism from United Kingdom traveler to Pakistan; or organism from the global collection), plasmid content (p60006, described in this study, or pHCM2 cryptic plasmid), presence of chromosomal AMR cassette (integrated composite transposon), and quinolone resistance-determining region (QRDR) mutations according to the color key.

We determined the genotype of the samples according to the typing framework described in the work of Wong et. al. (9). All of the XDR isolates and 11 out of 12 of the contextual (ceftriaxone-sensitive) isolates belonged to the 4.3.1 (H58) clade. In order to determine the phylogenetic relationship of the Sindh, Pakistan, isolates within the H58 lineage, we constructed a maximum-likelihood phylogenetic tree with H58 isolates from the previously analyzed global *S*. Typhi collection (3). The XDR isolates and four of the contextual Sindh isolates were located on a single branch separated from the other H58 isolates by 17 single nucleotide polymorphisms (SNPs) (Fig. 1 and S2). On this branch, the XDR isolates formed a tight cluster with 6 SNPs unique to the XDR cluster. Within the XDR cluster, there were 17 SNPs in total and a maximum pairwise distance between isolates of only four SNPs. The lack of diversity and highly clonal nature reflect the short sampling period and are indicative of an outbreak. Thus, there has been remarkably little genetic change during transmission, and this may indicate a single point source or origin.

10.1128/mBio.00105-18.2 FIG S2  Phylogenetic tree of global H58 isolates, including Sindh, Pakistan, XDR samples. A maximum-likelihood phylogenetic tree of 98 Sindh, Pakistan, H58 isolates; 1 United Kingdom traveler isolate; and 853 global H58 isolates inferred from 1,920 SNPs using 22420_1_10_Pak60006_2016 as a reference rooted on nearest neighbor 10060_5_62_Fij107364_2012. Download FIG S2, PDF file, 1.3 MB. Copyright © 2018 Klemm et al. 2018 Klemm et al. This content is distributed under the terms of the Creative Commons Attribution 4.0 International license.

The phylogenetic branch consisted of isolates from a geographic region covering Pakistan, India, Bangladesh, Iraq, and Palestine. Isolates without the p60006 plasmid on this branch have been circulating in Pakistan since at least 2010. Therefore, the XDR clone is likely derived from an endemic Pakistan clone that recently acquired ceftriaxone resistance.

**FIG 2:**
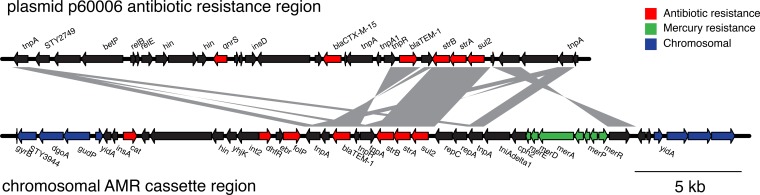
XDR *S*. Typhi isolates from Sindh, Pakistan, contain antibiotic resistance genes on the plasmid and chromosome. Genetic map of regions on the plasmid and chromosome from 22420_1_10_Pak60006_2016 containing antibiotic resistance-associated features. Chromosomal antibiotic resistance region refers to the composite transposon observed previously in H58 isolates. Regions with BLAST identity of >99% are shown in gray. The figure was made using genoPlotR (34).

We next identified the antibiotic resistance genes carried by the isolates. Since antibiotic resistance in H58 is commonly found on a composite transposon that is either located on an IncHI1 plasmid or integrated into the chromosome at one of two sites (near *yidA* or *cyaA*) (3), we searched for this region. All of the XDR isolates had the H58-associated composite transposon antimicrobial resistance (AMR) cassette integrated into the chromosome at the *yidA* site (Fig. 1b and 2). The composite transposon contains genes that impart resistance to chloramphenicol (*catA1*), ampicillin (*bla*_TEM-1_), trimethoprim-sulfamethoxazole (*dfrA7*, *sul1*, and *sul2*), and streptomycin (*strA* and *strB*), all of which are present in the XDR samples (Fig. 2). Unlike most other H58 isolates, the Pakistan XDR isolates were additionally resistant to ceftriaxone and ciprofloxacin, and therefore, we sought to identify the genetic basis of this phenotype. We found that the XDR isolates harbored a *bla*_CTX-M-15_ extended-spectrum β-lactamase (ESBL) gene that mediates resistance to ceftriaxone. The high MIC of ciprofloxacin could be attributed to the combination of a single mutation in *gyrA* (S83F) and the acquisition of a *qnrS* gene. The contextual isolates from Sindh, Pakistan, which have an intermediate-susceptibility phenotype for ciprofloxacin have the single *gyrA* (S83F) mutation but not the *qnrS* gene (Fig. 1b; Table S1).

The *bla*_CTX-M-15_ and *qnrS* genes were carried on an IncY plasmid specific to the XDR isolates in this branch that we named p60006. The antibiotic resistance loci from the plasmid and the chromosome of the XDR isolate 60006 are shown in Fig. 2. Several genes and regions of DNA sequence homology were shared between the plasmid and the chromosomally integrated AMR cassette, including a Tn*6029* transposon with *bla*_TEM-1_, *strA*, *strB*, and *sul2* genes. Plasmid p60006 also contained the complete VirB/Tra locus for self-transmissible plasmid conjugation.

**FIG 3:**
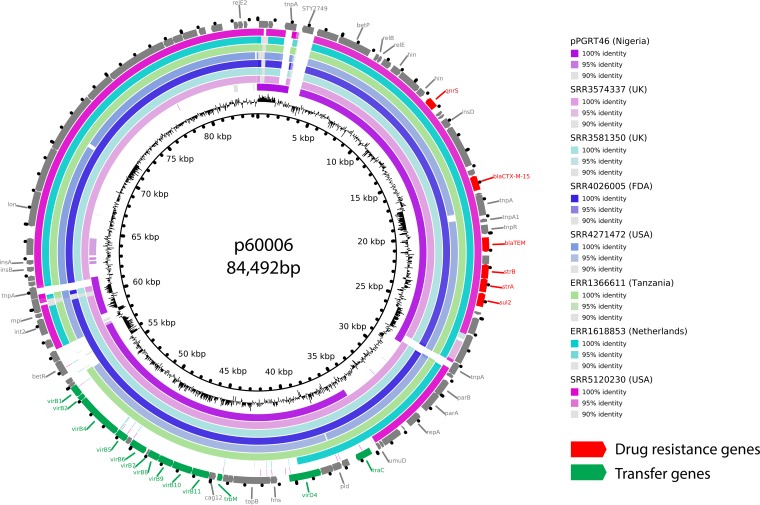
The plasmid from XDR *S*. Typhi isolates from Sindh, Pakistan, is highly similar to global *E. coli*-associated plasmids. Plasmid p60006 was compared to representative isolates from each study described in Table S2 using BRIG (35). The innermost circle shows GC content, and the outermost circle shows the gene map, colored red for drug resistance or green for transfer activity.

Plasmids are known to move through bacterial populations and transfer antibiotic resistance between species (10). Consistent with this, plasmid p60006 exhibited high DNA sequence identity to a previously sequenced plasmid, pPGRT46, isolated from a Nigerian *E. coli* isolate (11). Both plasmids shared the AMR and transfer loci in synteny (Fig. S3). Further investigation of public DNA databases identified a further 20 *E. coli* isolates from seven different studies that potentially contained a plasmid sequence similar to p60006 (Table S2). Remarkably, these organisms were isolated from six different countries on four different continents and are from environmental, animal, and human sources (12–14). We next mapped the raw sequencing reads from these *E. coli* strains to p60006 to create assembled sequences that were compared using BLAST. The *E. coli* DNA sequences exhibited high sequence identity to p60006 (Fig. 3). Some genomes were lacking a short region containing the *betR* gene involved in betaine transport and osmoregulation, while others lacked the plasmid transfer locus; however, all contained the AMR locus and the majority of the plasmid. p60006 has acquired an additional gene, STY2749, that was not found in the *E. coli* genomes. STY2749 is a hypothetical *S*. Typhi gene of unknown function, not present on the chromosome of the 60006 XDR isolate. We hypothesize that this plasmid originated in *E. coli* and was acquired by *S*. Typhi in Pakistan prior to the emergence and spread of the XDR clone.

10.1128/mBio.00105-18.3 FIG S3  p60006 and pPGRT46 share conserved regions of high sequence similarity. Plasmid p60006 (from XDR *S*. Typhi in Sindh, Pakistan) was compared by BLAST to plasmid pPGRT46 (from *E. coli* in Nigeria). Regions of BLAST identity of >99% are shown in gray. Antibiotic resistance genes are in red, and transfer genes are in green. Figure made with genPlotR (34). Download FIG S3, PDF file, 0.7 MB. Copyright © 2018 Klemm et al. 2018 Klemm et al. This content is distributed under the terms of the Creative Commons Attribution 4.0 International license.

10.1128/mBio.00105-18.5 TABLE S2  Samples with high sequence similarity to plasmid p60006. Download TABLE S2, XLSX file, 0.03 MB. Copyright © 2018 Klemm et al. 2018 Klemm et al. This content is distributed under the terms of the Creative Commons Attribution 4.0 International license.

During our analysis, we were alerted by Public Health England that a ceftriaxone-resistant *S*. Typhi sample had been isolated from a patient in the United Kingdom who had recently traveled from Pakistan. We compared the genome sequences and found that this isolate belonged to the same phylogenetic cluster of emerging XDR isolates from Sindh, Pakistan (Fig. 1b).

## DISCUSSION

We report the emergence of an XDR *S*. Typhi clone resistant to chloramphenicol, ampicillin, trimethoprim-sulfamethoxazole, fluoroquinolones, and third-generation cephalosporins that spread throughout the region in Pakistan and as far as the United Kingdom. We propose that *S*. Typhi resistant to five antibiotics should be referred to as “extensively drug-resistant” (XDR) according to the similar nomenclature used for *Mycobacterium tuberculosis* and other bacterial pathogens (15). Whole-genome sequencing enabled a thorough genetic characterization of the emergent clone. The presence of the same phylogenetic lineage in Pakistan prior to 2016 demonstrates that it was likely not the result of importation from outside Pakistan. The evidence presented here suggests that an endemic MDR H58 clone acquired an ESBL-encoding AMR plasmid, potentially from an *E. coli* strain or another enteric bacterial donor. The resultant XDR *S*. Typhi then underwent a clonal expansion during its emergence and spread. Determining the sequence of the plasmid enabled us to infer that plasmids with similar gene structures are omnipresent in diverse geographic settings.

The emergence of this clone marks a sentinel event in the evolution of antibiotic resistance in *S*. Typhi: previous reports of XDR typhoid were sporadic, isolated cases, whereas this was a large-scale emergence of temporally clustered cases that spread throughout the region and was even carried to the United Kingdom. To our knowledge, there have been 17 reports in the literature of *S*. Typhi with sporadic third-generation cephalosporin resistance (see Table S3 in the supplemental material). Of those, only four individual cases (single patients) have reported *S*. Typhi that was also both MDR and fluoroquinolone resistant (16–20). These cases originated from Iraq, Bangladesh, India, and Pakistan. The Bangladesh and Iraq isolates also harbor *bla*_CTX-M_ ESBL genes, but the Iraq isolate reportedly had an IncN plasmid, which differs from the IncY plasmid identified in this study. A draft genome of the Pakistan case from Rawalpindi has been released (20), and it harbors the same plasmid that we identified in the Sindh, Pakistan, cases.

10.1128/mBio.00105-18.6 TABLE S3  Published studies reporting third-generation-cephalosporin-resistant *S*. Typhi. Download TABLE S3, DOCX file, 0.03 MB. Copyright © 2018 Klemm et al. 2018 Klemm et al. This content is distributed under the terms of the Creative Commons Attribution 4.0 International license.

Typhoid fever is a reportable illness in the Sindh province of Pakistan. The cases identified were reported to the Sindh health authorities with a special note to indicate the emergence of ceftriaxone resistance. The sudden emergence and rapid spread of resistant isolates underline the importance of AMR surveillance for typhoid and other enteric Gram-negative bacteria and highlight the inadequacy of relying solely on non-culture-based methods for diagnosis of typhoid (such as Widal and Typhidot tests), which do not provide susceptibility results. In view of the emergence of ceftriaxone resistance in *S*. Typhi, culture- and sensitivity-guided treatment becomes imperative as empirical treatment with ceftriaxone is no longer reliable in the region. Following antibiotic resistance testing, cases were effectively treated with azithromycin and meropenem, resulting in recovery by most patients. Immediate control measures instituted by the government included education of the public and emphasis on hygiene and food safety. However, a mass vaccination campaign could not immediately be undertaken to prevent the spread of this highly resistant clone.

The emergence and spread of XDR *S*. Typhi in Sindh, Pakistan, are a startling demonstration of how a ubiquitous antibiotic resistance plasmid can be acquired by MDR *S*. Typhi, rendering it XDR and further narrowing treatment options. The fact that this clone reached as far as the United Kingdom showed the direct impact that regional clones can have on the health of other countries. Antibiotics save millions of lives annually, but the apparent ease and rapidity by which life-threatening bacteria such as *S*. Typhi can develop resistance severely limit their efficacy. Our data suggest that better strategies against typhoid are warranted, such as the introduction of preventive measures, including vaccines and improved sanitation.

## MATERIALS AND METHODS

### Strains, identification, and susceptibilities.

Blood cultures submitted to the Aga Khan University clinical microbiology laboratory (November 2016 to September 2017) grew *S*. Typhi that demonstrated high MICs against ceftriaxone and cefotaxime (>64 µg/ml). MICs were confirmed by two methods, Etest and Vitek 2 (bioMérieux). The identification of *S*. Typhi was confirmed by the API 20E test (bioMérieux) and agglutination with genus- and serotype-specific antisera (Salmonella poly antiserum A-I [Difco], Salmonella O antiserum [Difco], and Salmonella Vi antiserum [Difco]).

### Illumina sequencing.

Extracted DNA (prepared with the Promega Wizard genomic purification kit) was used to make multiplex libraries with a 500-bp insert size, which were prepared using unique index tags and sequenced to generate 250-base-paired-end reads. Cluster formation, primer hybridization, and sequencing reactions were based on reversible terminator chemistry using the Illumina HiSeq 2500 System.

### Nanopore sequencing.

DNA was quality checked using Qubit (Thermo Fisher) using the Broad Range kit and the Agilent TapeStation, which registered a peak fragment size of 58 kb. Oxford nanopore sequencing was carried out using flow cell Flo-MIN107 (R9.5 nanopore) and the rapid barcoding kit SQK-RBK001, with the barcode NB01 chosen for this isolate. Barcoding was used to enable exclusion of reads from this first isolate in subsequent data, in the event that this flow cell was reused for other isolates at a later date. MinKNOW version 1.6.11 was used with local 1D base calling enabled. The sequencing run was stopped after 23 hours as sufficient data had been obtained.

### Computing infrastructure.

All of the analysis was performed on the Wellcome Trust Sanger Institute’s computing cluster running Linux Ubuntu 12.04 on servers with 32 CPUs and 256 GB of RAM. Only open-source software was utilized, allowing for transparent reproducibility.

### Genotyping.

Genotyping was done according to the framework described in reference 9 using the genotyphi code (https://github.com/katholt/genotyphi).

### Reference genome assembly.

A hybrid assembly was performed using both the short- and long-read sequencing data for BL60006 using Unicycler (v0.4.0) (21). A single circularized chromosome and a single plasmid were assembled. As a region was shared between the plasmid and chromosome, further sequencing using the PacBio RSII was required to create a fully circularized plasmid assembly. The PacBio reads were assembled using the SMRT analysis pipeline (v2.3.0), followed by polishing with Unicycler and Pilon (v1.19) (22) using the corresponding Illumina short reads. Finally, the plasmid was circularized with Circlator (v1.4.0) (23). The final assembled chromosome consists of 4,733,003 bases, and the plasmid consists of 84,492 bases.

### Masking duplicated regions in reference genome.

Variant calling using short-read sequences is more erroneous around regions where there are duplicated sequences in the genome. Blastn (v2.6.0) (24) is run on the reference genome against itself, and any BLAST hits where the length is greater than 300 (66% of the fragment size) with identity greater than 98% are kept. Self-matches were ignored, and overlapping regions were resolved into contiguous blocks. These coordinates were then masked out in subsequent analysis with N’s to avoid calling variants at these positions.

### Mapping, SNP calling, and pseudogenome generation.

For each sample, sequence reads were mapped using SMALT (v0.7.4) (25) against a given reference to produce a BAM file. SMALT was used to index the reference using a kmer size of 20 and a step size of 13, and the reads were aligned using default parameters but with the maximum insert size (1,500) set as 3 times the mean fragment size of the sequencing library. PCR duplicate reads were identified using Picard (v1.92) (http://broadinstitute.github.io/picard/) and flagged as duplicates in the BAM file. Variation detection was performed using SAMtools mpileup v0.1.19 (26) with parameters “-d 1000 -DSugBf” and bcftools v0.1.19 to produce a BCF file of all variant sites. The option to call genotypes at variant sites was passed to the bcftools call. All bases were filtered to remove those with uncertainty in the base call. The bcftools variant quality score was required to be greater than 50 (quality of >50), and mapping quality had to be greater than 30 (map quality of >30). If not all reads gave the same base call, the allele frequency, as calculated by bcftools, was required to be either 0, for bases called the same as the reference, or 1, for bases called as an SNP (af1 of >0.95). The majority base call was required to be present in at least 75% of reads mapping at the base (ratio of >0.75), and the minimum mapping depth required was 4 reads, at least two of which had to map to each strand (depth of >4, depth strand of >2). Finally, strand bias was required to be less than 0.001, map bias was required to be less than 0.001, and tail bias was required to be less than 0.001. If any of these filters were not met, the base was called as uncertain. A pseudogenome was constructed by placing the base call at each site (variant and nonvariant) in the BCF file into the reference genome as a substitute, and any site called as uncertain was replaced with an N. Insertions with respect to the reference genome were ignored and deletions with respect to the reference genome were filled with N’s in the pseudogenome to keep it aligned and the same length as the reference genome used for read mapping. All of this analysis was performed within an open-source pipeline (https://github.com/sanger-pathogens/vr-codebase).

### Recombination.

Horizontal recombination must be excluded from each multiple-FASTA alignment before building a phylogenetic tree, as it is unrelated to the phylogenetic evolution of the samples. An alignment was provided to Gubbins (v1.4.10) (27) using default parameters. The resulting masked alignment was then used as input for phylogenetic tree construction.

### Phylogenetic tree construction.

Phylogenetic trees were constructed using RAxML (v8.2.8) (28) with multi-FASTA alignments of nucleotide sequences as input, generated as described previously. The gamma general time reversible (GAMMAGTR) model was used in each case with 100 random bootstraps. Trees were outputted in NEWICK format.

### Antimicrobial resistance gene identification and plasmid typing.

The FASTQ files for each sample were provided to ARIBA (v2.10.0) to detect AMR genes. The CARD database (v1.1.8) (29) was utilized for AMR detection. Point mutations in the QRDR were determined using WGSA (https://www.wgsa.net). Plasmid replicons were identified using ARIBA and the PlasmidFinder database (30).

### *E. coli* plasmid sequences and comparison.

Fragments of the p60006 plasmid were searched against a Coloured Bloom Graph of the entire set of bacteria in the ENA (https://github.com/Phelimb/cbg), which identified 20 *E. coli* samples as potentially containing a similar plasmid sequence. The raw reads for each were downloaded from the ENA in FASTQ format, with each corresponding to an Illumina paired-end sequencing experiment. The raw reads were filtered with Trimmomatic (v0.32) (31) to remove adapter sequences and low-quality bases. The p60006 plasmid was used as a reference genome for generating a pseudogenome multiple-FASTA alignment (as described previously) with the filtered FASTQ files. SNPs were identified using SNP-sites (v2.3.2) (32), and each variant was visually confirmed using Artemis (v16.0.18) (33) and the associated BAM file. Individual assemblies were compared to the p60006 plasmid using BRIG with default BLAST parameters.

### Accession number(s).

The accession numbers for the assembled chromosome of isolate 22420_1_10_Pak60006_2016 and its plasmid are LT882486 and LT906492, respectively. Sequence data were submitted to the European Nucleotide Archive, and accession numbers are indicated in Table S1.
