# Supplementary figures and images for: Emergence of an Extensively Drug-Resistant Salmonella enterica Serovar Typhi Clone Harboring a Promiscuous Plasmid Encoding Resistance to Fluoroquinolones and Third-Generation Cephalosporins
Source: mBio. 2018 Feb 20;9(1):e00105-18. doi: 10.1128/mBio.00105-18 (PMC5821095; doi:10.1128/mBio.00105-18)

a.

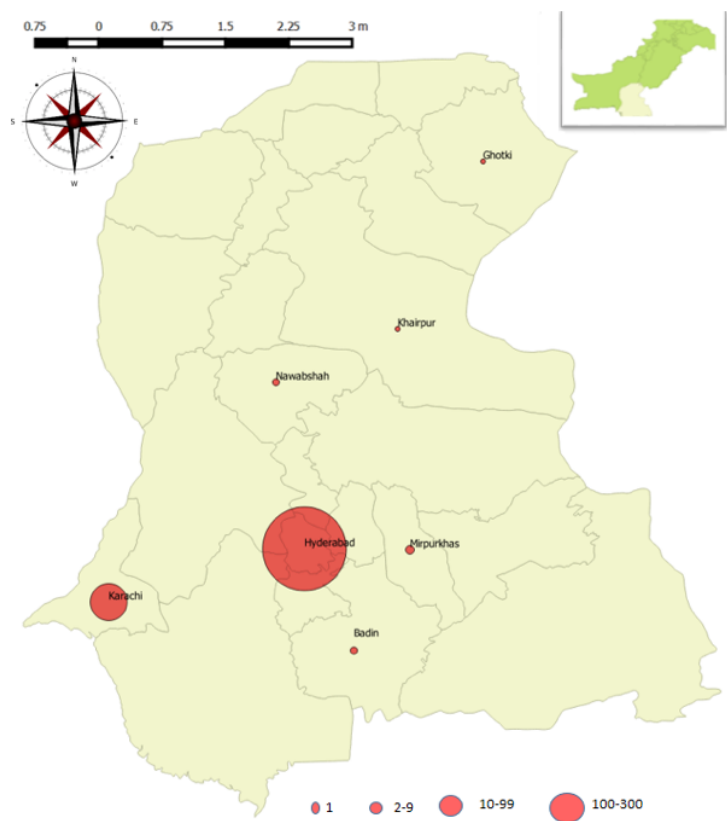

b.

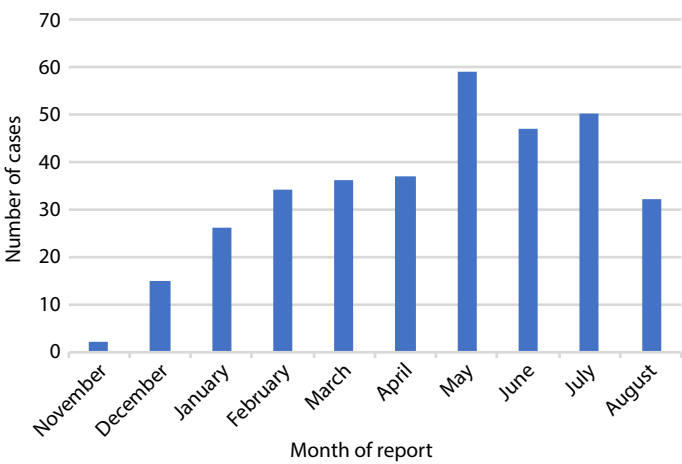

c.

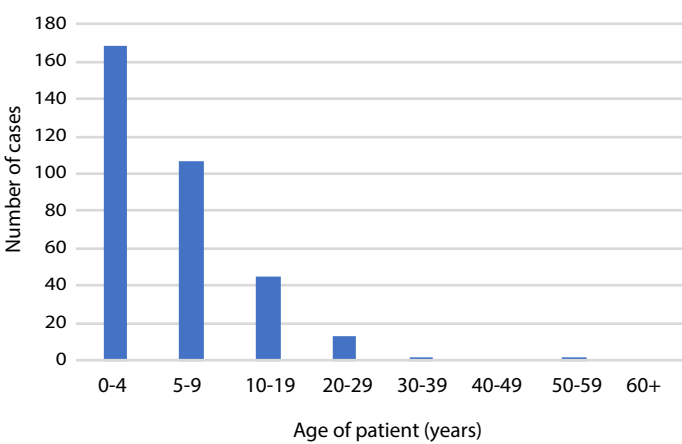

Supplement: FIG S1 [file mbo001183737sf1.pdf]

p60006

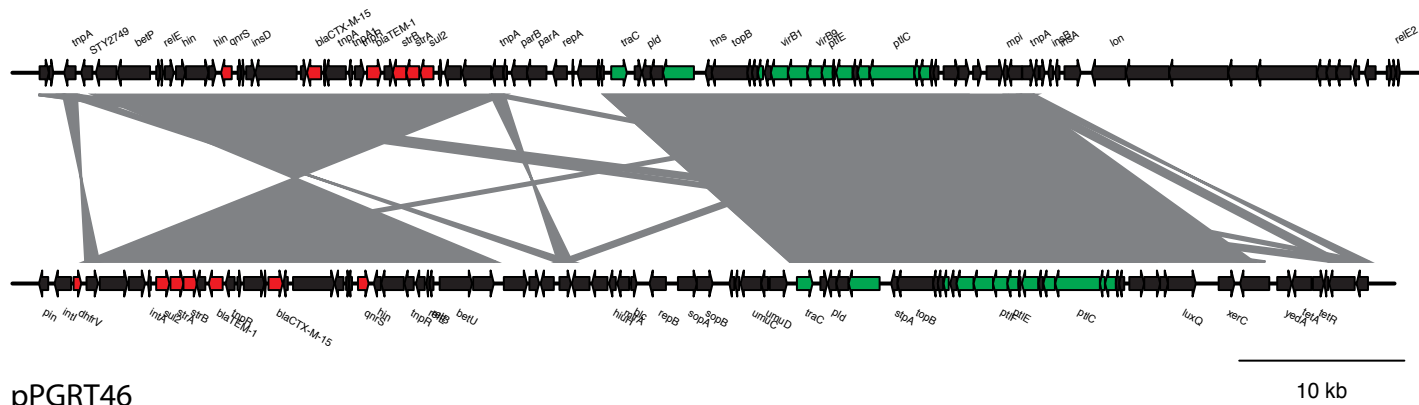

Supplement: FIG S3 [file mbo001183737sf3.pdf]
