# Supplementary material for: Emergence of an Extensively Drug-Resistant Salmonella enterica Serovar Typhi Clone Harboring a Promiscuous Plasmid Encoding Resistance to Fluoroquinolones and Third-Generation Cephalosporins
Source: mBio. 2018 Feb 20;9(1):e00105-18. doi: 10.1128/mBio.00105-18 (PMC5821095; doi:10.1128/mBio.00105-18)
Supplement: FIG S2 [file mbo001183737sf2.pdf]

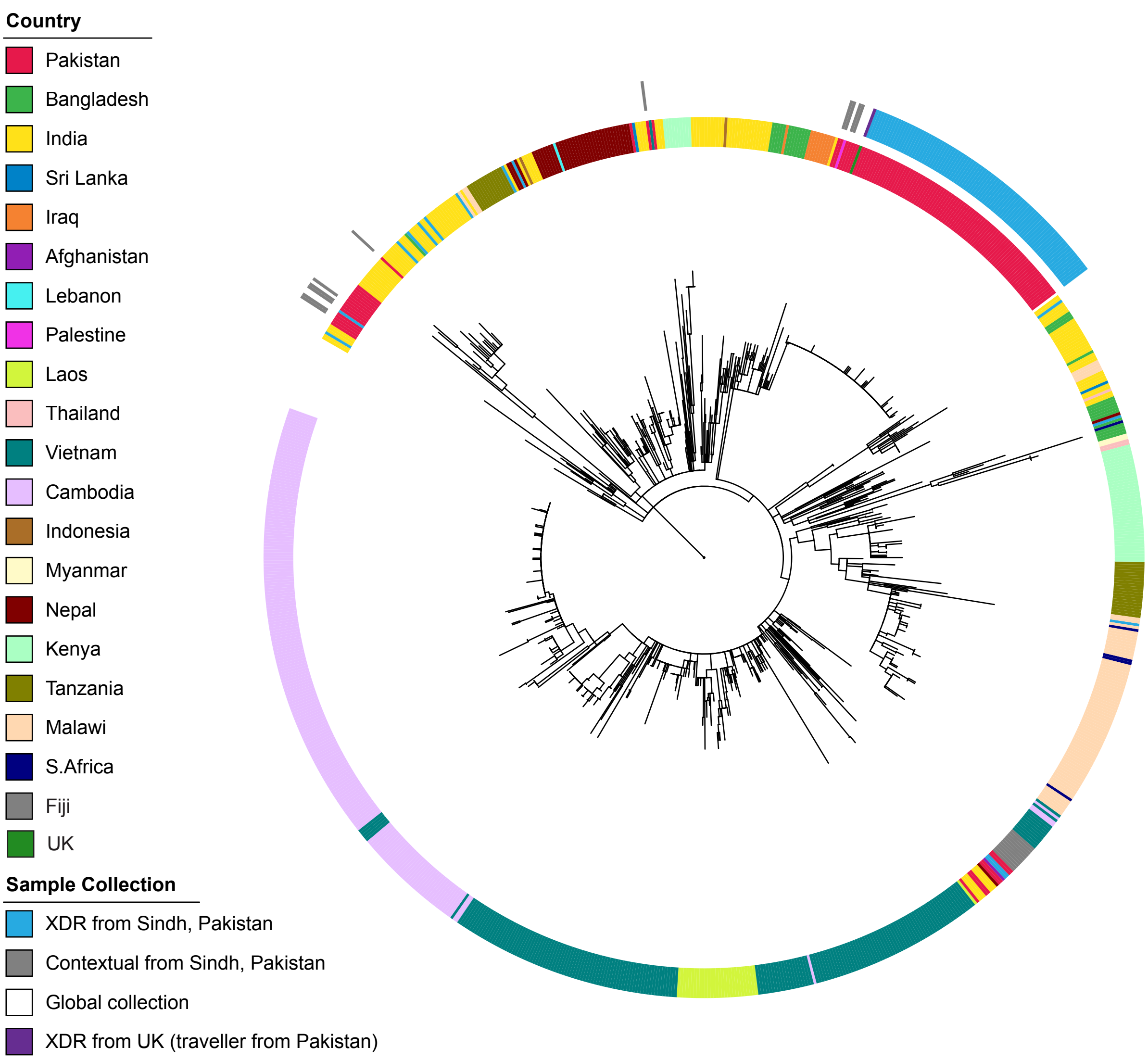
Country
Pakistan
Bangladesh
India
Sri Lanka
Iraq
Afghanistan
Lebanon
Palestine
Laos
Thailand
Vietnam
Cambodia
Indonesia
Myanmar
Nepal
Kenya
Tanzania
Malawi
S.Africa
Fiji
UK
Sample Collection
XDR from Sindh, Pakistan
Contextual from Sindh, Pakistan
Global collection
XDR from UK (traveller from Pakistan)
