# Supplementary material for: Emergence of an Extensively Drug-Resistant Salmonella enterica Serovar Typhi Clone Harboring a Promiscuous Plasmid Encoding Resistance to Fluoroquinolones and Third-Generation Cephalosporins
Source: mBio. 2018 Feb 20;9(1):e00105-18. doi: 10.1128/mBio.00105-18 (PMC5821095; doi:10.1128/mBio.00105-18)
Supplement: TABLE S3 [file mbo001183737st3.docx]

**Table S3**

**Published studies reporting third-generation cephalosporin-resistant *S*. Typhi**

| **Paper** | **Country of origin (report)** | **Year** | **Number of cases** | **ESBL gene** | **Plasmid** |
| --- | --- | --- | --- | --- | --- |
| Saha *et. al.*(1) | Bangladesh | 1999 | 1 | unknown | unknown |
| Rotimi *et. al*.(2) | Kuwait and UAE | 2003-2006 | 3 | *bla*_CTX-M-15_ | unknown |
| Al Naiemi *et. al*.(3) | Philippines (Netherlands) | 2007 | 1 | *bla*_SHV-12_ | unknown |
| Hendriksen *et. al*.(4) | Philippines (Norway) | 2007 | 1 | *bla*_SHV-12_ | IncHI2 |
| Morita *et. al*.(5) | Japan | 2008 | 1 | *bla*_CTX-M-15_ | unknown |
| Pfeifer *et. al*.(6) | Iraq (Germany) | 2008 | 1 | *bla*_CTX-M-15_ | IncN |
| Kumarasamy *et. al*.(7) | India | 2009 | 1 | *bla*_CMY-2_ | IncA/C |
| Gokul *et. al*.(8) | India | 2009 | 1 | *bla*_ACC-1_ | unknown |
| Qamar *et. al*.(9) | Pakistan | 2009-2011 | 2 | unknown | unknown |
| Akinyemi *et. al.*(10) | Nigeria | 2010-2011 | 9 | *bla*_CTX-M-I_ | unknown |
| Jain *et. al*.(11) | India | 2012 | 4 | unknown | unknown |
| Ahmed *et. al*.(12) | Bangladesh | 2012 (?) | 1 | *bla*_CTX-M_ | unknown |
| Gonzalez-Lopez *et. al*.(13) | Guatemala (Spain) | 2013 | 1 | *bla*_CTX-M-15_ | IncL/M |
| Phoba *et. al.(14)* | DRC | 2015 | 1 | *bla*_CTX-M-15_ | IncY |
| Rodrigues *et. al*.(15) | India | 2016 | 4 | *bla*_SHV-12_ and *bla*_CMY-2_ | IncX3 and IncA/C2 |
| Munir *et. al*.(16) and  Gul *et. al*.(17) | Pakistan | 2016 (?) | 1 | *bla*_CTX-M-15_ | p60006 |
| Kleine *et. al.*(18) | India (Germany) | 2016 (?) | 1 | *bla*_SHV-12_ | unknown |

1. Saha SK, Talukder SY, Islam M, Saha S. 1999. A highly ceftriaxone-resistant Salmonella typhi in Bangladesh. Pediatr Infect Dis J 18:387.

2. Rotimi VO, Jamal W, Pal T, Sovenned A, Albert MJ. 2008. Emergence of CTX-M-15 type extended-spectrum beta-lactamase-producing Salmonella spp. in Kuwait and the United Arab Emirates. J Med Microbiol 57:881-6.

3. Al Naiemi N, Zwart B, Rijnsburger MC, Roosendaal R, Debets-Ossenkopp YJ, Mulder JA, Fijen CA, Maten W, Vandenbroucke-Grauls CM, Savelkoul PH. 2008. Extended-spectrum-beta-lactamase production in a Salmonella enterica serotype Typhi strain from the Philippines. J Clin Microbiol 46:2794-5.

4. Hendriksen RS, Leekitcharoenphon P, Mikoleit M, Jensen JD, Kaas RS, Roer L, Joshi HB, Pornruangmong S, Pulsrikarn C, Gonzalez-Aviles GD, Reuland EA, Al Naiemi N, Wester AL, Aarestrup FM, Hasman H. 2015. Genomic dissection of travel-associated extended-spectrum-beta-lactamase-producing Salmonella enterica serovar typhi isolates originating from the Philippines: a one-off occurrence or a threat to effective treatment of typhoid fever? J Clin Microbiol 53:677-80.

5. Morita M, Takai N, Terajima J, Watanabe H, Kurokawa M, Sagara H, Ohnishi K, Izumiya H. 2010. Plasmid-mediated resistance to cephalosporins in Salmonella enterica serovar Typhi. Antimicrob Agents Chemother 54:3991-2.

6. Pfeifer Y, Matten J, Rabsch W. 2009. Salmonella enterica serovar Typhi with CTX-M beta-lactamase, Germany. Emerg Infect Dis 15:1533-5.

7. Kumarasamy K, Krishnan P. 2012. Report of a Salmonella enterica serovar Typhi isolate from India producing CMY-2 AmpC beta-lactamase. J Antimicrob Chemother 67:775-6.

8. Gokul BN, Menezes GA, Harish BN. 2010. ACC-1 beta-Lactamase-producing Salmonella enterica Serovar Typhi, India. Emerg Infect Dis 16:1170-1.

9. Qamar FN, Azmatullah A, Kazi AM, Khan E, Zaidi AK. 2014. A three-year review of antimicrobial resistance of Salmonella enterica serovars Typhi and Paratyphi A in Pakistan. J Infect Dev Ctries 8:981-6.

10. Akinyemi KO, Iwalokun BA, Alafe OO, Mudashiru SA, Fakorede C. 2015. bla CTX-M-I group extended spectrum beta lactamase-producing Salmonella typhi from hospitalized patients in Lagos, Nigeria. Infect Drug Resist 8:99-106.

11. Jain S, Das Chugh T. 2013. Antimicrobial resistance among blood culture isolates of Salmonella enterica in New Delhi. J Infect Dev Ctries 7:788-95.

12. Ahmed D, Hoque A, Mazumder R, Nahar K, Islam N, Gazi SA, Hossain MA. 2012. Salmonella enterica serovar Typhi strain producing extended-spectrum beta-lactamases in Dhaka, Bangladesh. J Med Microbiol 61:1032-3.

13. Gonzalez-Lopez JJ, Piedra-Carrasco N, Salvador F, Rodriguez V, Sanchez-Montalva A, Planes AM, Molina I, Larrosa MN. 2014. ESBL-producing Salmonella enterica serovar Typhi in traveler returning from Guatemala to Spain. Emerg Infect Dis 20:1918-20.

14. Phoba M-F, Barbé B, Lunguya O, Masendu L, Lulengwa D, Dougan G, Wong VK, Bertrand S, Ceyssens P-J, Jacobs J, Van Puyvelde S, Deborggraeve S. 2017. Salmonella enterica serovar Typhi Producing CTX-M-15 Extended Spectrum β-Lactamase in the Democratic Republic of the Congo. Clinical Infectious Diseases 65:1229-1231.

15. Rodrigues C, Kapil A, Sharma A, Devanga Ragupathi NK, Inbanathan FY, Veeraraghavan B, Kang G. 2017. Whole-Genome Shotgun Sequencing of Cephalosporin-Resistant Salmonella enterica Serovar Typhi. Genome Announc 5.

16. Munir T, Lodhi M, Ansari JK, Andleeb S, Ahmed M. 2016. Extended Spectrum Beta Lactamase producing Cephalosporin resistant Salmonella Typhi, reported from Rawalpindi, Pakistan. J Pak Med Assoc 66:1035-6.

17. Gul D, Potter RF, Riaz H, Ashraf ST, Wallace MA, Munir T, Ali A, Burnham CA, Dantas G, Andleeb S. 2017. Draft Genome Sequence of a Salmonella enterica Serovar Typhi Strain Resistant to Fourth-Generation Cephalosporin and Fluoroquinolone Antibiotics. Genome Announc 5.

18. Kleine CE, Schlabe S, Hischebeth GTR, Molitor E, Pfeifer Y, Wasmuth JC, Spengler U. 2017. Successful therapy of a multi-resistant EBSL (SHV-12)-producing and fluoroquinolone-resistant Salmonella enterica subsp. enterica serovar Typhi infection using combination therapy of meropenem and fosfomycin. Clin Infect Dis doi:10.1093/cid/cix652.
